# Biomedical Applications of Reactive Oxygen Species Generation by Metal Nanoparticles

**DOI:** 10.3390/ma14010053

**Published:** 2020-12-24

**Authors:** Roberto Canaparo, Federica Foglietta, Tania Limongi, Loredana Serpe

**Affiliations:** 1 Department of Drug Science and Technology, University of Torino, Via Pietro Giuria 13, 10125 Torino, Italy; roberto.canaparo@unito.it (R.C.); federica.foglietta@unito.it (F.F.); 2 Department of Applied Science & Technology, Politecnico di Torino, Corso Duca degli Abruzzi 24, 10129 Torino, Italy; tania.limongi@polito.it

**Keywords:** metal nanoparticles, iron oxide nanoparticles, silver nanoparticles, gold nanoparticles, titanium dioxide nanoparticles, zinc nanoparticles, reactive oxygen species, photodynamic therapy, photothermal therapy, sonodynamic therapy

## Abstract

The design, synthesis and characterization of new nanomaterials represents one of the most dynamic and transversal aspects of nanotechnology applications in the biomedical field. New synthetic and engineering improvements allow the design of a wide range of biocompatible nanostructured materials (NSMs) and nanoparticles (NPs) which, with or without additional chemical and/or biomolecular surface modifications, are more frequently employed in applications for successful diagnostic, drug delivery and therapeutic procedures. Metal-based nanoparticles (MNPs) including metal NPs, metal oxide NPs, quantum dots (QDs) and magnetic NPs, thanks to their physical and chemical properties have gained much traction for their functional use in biomedicine. In this review it is highlighted how the generation of reactive oxygen species (ROS), which in many respects could be considered a negative aspect of the interaction of MNPs with biological matter, may be a surprising nanotechnology weapon. From the exchange of knowledge between branches such as materials science, nanotechnology, engineering, biochemistry and medicine, researchers and clinicians are setting and standardizing treatments by tuning ROS production to induce cancer or microbial cell death.

## 1. Introduction

Nanoscience refers to the study and application of tiny materials with dimensions equal to or less than 100 nm of which many other fields, such as material science, engineering, physics, chemistry, biology and medicine, can take advantage. One of the most active areas of research in this field is the study and the development of nanostructured materials (NSMs) and nanoparticles (NPs) [1].

NSMs and NPs have unique tunable physicochemical features such as catalytic activity, electrical and thermal conductivity, light absorption and scattering that, starting from bulk counterparts, allow enhanced performance to be exploited by many different areas such as food industry, agriculture, cosmetics and, of course, medicine [2]. In the latter area, NSMs and NPs have found suitable applications in fluorescent biological labeling [3,4], pathogen detection [5], protein analysis [6], DNA structure probing [7], tissue engineering [8], separation and purification of cells and biological molecules [9], magnetic resonance imaging (MRI) contrast enhancement [10], drug and gene delivery [11,12].

Particularly, in more recent decades, NPs have been successfully used in the clinic as effective tools for alternative therapy such as photodynamic therapy (PDT) [13,14,15], high-intensity focused ultrasound therapy (HIFU) [16], photothermal therapy (PPT) [17] and sonodynamic therapy (SDT) [18,19,20,21]. The ever-increasing success of these therapies is due to their ability to induce the death of prokaryotic and eukaryotic cells through key cellular mechanisms such as that of induction of NP-mediated reactive oxygen species (ROS) generation [22]. Some NPs, once released into the body through different internalization methods such as oral, parenteral, inhalation administration and skin adsorption, can affect redox homeostasis both by generating ROS or lessening scavenging pathways [22,23].

### 1.1. ROS Generation and Oxidative Stress

Reactive oxygen species, key signaling molecules during cell signaling and homeostasis, are produced in cells by oxidases, originating from the excitation and univalent reduction of the molecular oxygen, which leads to the generation of hydroxyl radicals, superoxide anion and hydrogen peroxide [24]. Briefly, molecular oxygen generates superoxide anion, the primary ROS, via reduction of one electron catalyzed by nicotinamide adenine dinucleotide phosphate (NADPH) oxidase. Further reduction of oxygen may either lead to hydrogen peroxide or hydroxyl radicals via dismutation and metal-catalyzed Fenton reaction, respectively [25,26]. Some of the endogenous sources of ROS include mitochondrial respiration, inflammatory response, microsomes and peroxisomes. However, the occurrence of free radicals from essential byproducts of mitochondrial respiration and transition metal ion-catalyzed Fenton-type reactions mainly can regulate many signal transduction paths in a dose-dependent way. While low or medium ROS levels raise mitogenic signaling via reversible oxidations, high ROS levels lead to nucleic acids and lipid oxidation and peroxidation, resulting in cellular apoptosis and necrosis phenomena [25,27,28,29,30].

**Figure 1:**
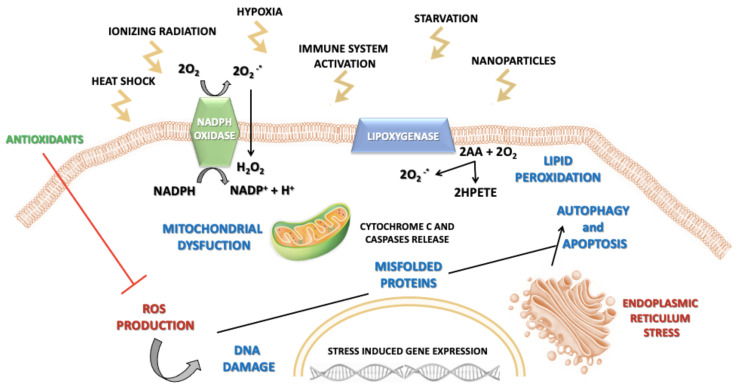
Schematic illustration of various triggers responsible for reactive oxygen species (ROS) generation and ROS-induced pathways leading to cell damage.

Along with free-radical and non-free radical oxygen-containing molecules, there are also reactive nitrogen, iron (Fe), copper (Cu), and sulfur species which could attribute to increased ROS formation and oxidative stress and thus impairing the redox balance [31,32]. In this regard, the appropriate physiological level of ROS is managed by antioxidant molecules such as glutathione (GSH), vitamin E, ascorbic acid, flavonoids and by detoxifying enzymes, such as catalase (CAT), glutathione peroxidase (GPX) and superoxide dismutase (SOD) [33]. According to this model, cells and tissues respond to increasing levels of oxidative stress via antioxidant enzyme systems. During conditions of mild oxidative stress, transcriptional activation of phase II antioxidant enzymes occurs via nuclear factor (erythroid- derived 2)-like 2 (Nrf2) induction. At an intermediate level, redox-sensitive mitogen-activated protein kinase (MAPK) and nuclear factor kappa-light-chain enhancer of activated B cells (NF-κB) cascades trigger a proinflammatory response. However, extremely toxic levels of oxidative stress result in mitochondrial membrane damage and electron chain dysfunction leading to cell death [24]. Therefore, perturbation of the normal redox state contributes to peroxide and free radical production that has adverse effects on cell components including proteins, lipids and DNA [34], leading to loss of cell growth, fibrosis and carcinogenesis [35,36,37] (Figure 1).

### 1.2. NPs-Induced Oxidative Stress

NPs of varying chemical composition such as metal oxides have been shown to induce oxidative stress and, in this regard, NPs have been reported to influence intracellular calcium concentrations, activate transcription factors and modulate cytokine production via generation of free radicals [22,25,38,39,40,41]. The main key factors involved in NP-induced ROS include: prooxidant functional groups on the reactive surface of NPs, active redox cycling on the surface of NPs due to transition metal-based NPs (MNPs), and particle-cell interactions. With regards to these key factors, several studies have shown the significance of reactive particle surfaces in ROS generation [25,39,42,43].

Free radicals are generated from the surface of NPs when both the oxidants and free radicals bind to the particle surface. Moreover, reduced particle size results in structural defects and can alter electronic properties on the NP surface, thereby creating reactive groups [44,45]. Within these reactive sites, the electron donor or acceptor interact with molecular oxygen to form superoxide anion which in turn can generate additional ROS via Fenton-type reactions [46]. For instance, NPs such as silica (Si) and zinc (Zn) with identical particle size and shape lead to diverse cytotoxicity responses due to their surface properties. Zinc oxide (ZnO), being more chemically active than silicon dioxide (SiO_2_), leads to increased superoxide anion formation, resulting in oxidative stress [47]. Moreover, the mechanism for NP-mediated ROS generation can be influenced by physicochemical features of NPs such as size, chemical structure, surface area and charge. Furthermore, transition metals such as Si, Zn, Cu, Fe, chromium (Cr) and vanadium (V) are associated with ROS generation through Fenton and Haber-Weiss reaction mechanisms [42]. In Fenton responses, a transition metal ion, reacting with hydrogen peroxide, yields hydroxyl radicals and an oxidized metal ion [26]. Metal-based NPs, such as Cu and Fe, affect oxidative stress by way of Fenton reactions. On the other hand, the Haber-Weiss reaction explains the generation of hydroxyl radicals via a reaction between hydrogen peroxide and oxidized metal ions [26,37,45]. Furthermore, cobalt (Co), Cr and V NPs can catalyze both Haber-Weiss and Fenton responses, considering that the Fenton reactions are also implicated in iron-oxide NPs (IONPs)-induced ROS generation processes [31]. Finally, some NPs promote the activation of intercellular radical-inducing systems such as the MAPK and NF-κB pathways [48].

In addition to the prooxidant effect of NPs, ROS are also induced endogenously where the mitochondrion is a major cell target for NP-induced oxidative stress. Specifically, once NPs gain access into the mitochondria, they stimulate ROS via impaired electron transport chain, structural damage, activation of NADPH-like enzyme systems and depolarization of the mitochondrial membrane [49,50].

### 1.3. NP-Induced Cell Death

Apoptosis has been implicated as a major mechanism of cell death caused by NP-induced oxidative stress [51,52]. Among the different apoptotic pathways, the intrinsic mitochondrial apoptotic pathway plays a major role in metal oxide NP-induced cell death, since mitochondria are one of the major target organelles for NP-induced oxidative stress [50]. High levels of ROS in the mitochondria can result in membrane phospholipid damage and in mitochondrial membrane depolarization [53]. A small proportion of electrons escapes the mitochondrial chain and interacts with molecular oxygen to form superoxide anion which later gives rise to hydrogen peroxide or partially reduces to damaging hydroxyl radicals. NPs can catalyze the superoxide anion generation either by blocking the electron transport chain or accelerating electron transfer to molecular oxygen [54,55]. Various metal oxide NPs including Zn, Cu, titanium (Ti), and Si elicit ROS-mediated cell death via mitochondrial dysfunction [56,57,58].

### 1.4. Introduction to Metal-Based NPs

Metal-based NPs have been used to revolutionize several fields including sensors, catalysis, optoelectronic materials and biomedical science. Such widespread applications are attributable to their electrochemical and physical properties, reflecting their small sizes and reactive surfaces. Their fixed particle mass, high aspect ratio and particle surface bioreactivity tailor them to meet the needs of specific applications. However, a high surface-to-volume ratio makes MNPs extremely reactive, particularly with regards to free radical generation [59,60]. Furthermore, nanoscale dimensions enhance cellular uptake and interaction with biological tissues. Metal-based NPs can generate free radicals via Fenton-type reactions that react with cellular macromolecules and induce oxidative stress [61].

In this review, the authors mainly discuss the role of MNPs in ROS generation for biomedical applications with special emphasis on highly selective approaches such as photodynamic, photothermal and sonodynamic therapy.

## 2. Metal-Based Nanoparticle Classes and Their Biomedical Applications

Among NPs we can identify two main groups: (i) organic NPs including liposomes, polymeric NPs, carbon-based NPs and dendrimers; and (ii) inorganic NPs including QDs, metal oxides and metal and magnetic NPs [62]. Referring to the metallic ones, it must be said that they can be designed and produced through different methods of synthesis and functionalization (Table 1). To improve their biotechnological drug delivery and theranostic applications, surface functionalization can be achieved with surfactants, polymers, drugs, oligonucleotides, peptides or antibodies.

A wide range of MNPs such as silver (AgNPs), gold (AuNPs), IONPs, zinc oxide (ZnONPs) and titanium dioxide (TiO_2_NPs) NPs have been mainly exploited after a whole series of optimizations and customizations for improving their applicability as therapeutic and/or diagnostic agents.

### 2.1. Iron-Oxide Magnetic NPs

**Figure 2:**
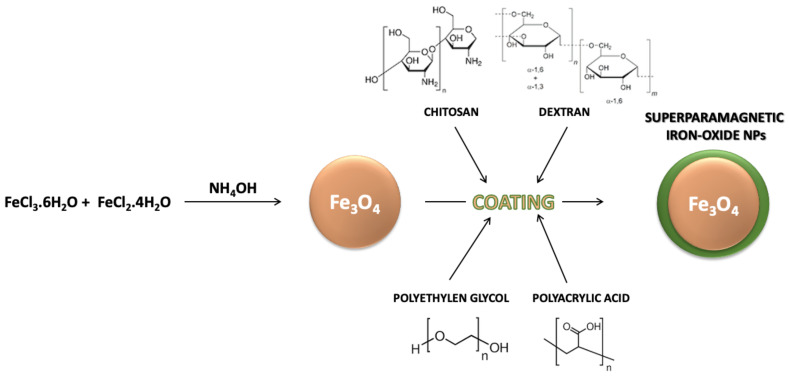
Iron NPs (Fe_3_O_4_ NPs) can be prepared by the chemical coprecipitation method and enriched with an external coating, including polyethylen glycol, polyacrylic acid, chitosan or dextran.

In biomedical applications, magnetic nanoparticles are characterized by small inorganic crystals (<20 nm diameter) of magnetic material that can result in a core-shell configuration by their coating with an organic layer or in a multicore-shell configuration by their embedding in an organic matrix [63]. The inorganic crystals of interest can consist of several materials with ferromagnetic or superparamagnetic behaviors (Figure 2). However, the so-called iron oxides such as magnetite and maghemite are the most prevalent materials [64]. In this case, the small size of the inorganic crystal enables the formation of particles with a single magnetic domain characterized by superparamagnetic properties. Moreover, as previously described, an organic shell is usually added to the magnetic NPs in order to give them colloidal stability in biological and aqueous fluids [64,65].

The ability of magnetic NPs to react with applied magnetic fields by translation (in magnetic field gradients), physical particle (in alternating and rotating fields) or internal dipole rotation (in alternating and rotating magnetic fields) shows great interests for biomedical applications [63]. The magnetic field energy can be locally converted into either thermal energy or mechanical forces and torques. Magnetic NPs are unique in possessing abilities that allow the external control of their movement and the use of their mechanical forces/torques on biological structures.

Thanks to the magnetic NP properties mentioned before, iron oxide NPs are studied for a broad range of biomedical applications such as MRI contrast agents [66], magnetically targeted drug delivery [67], magnetically assisted gene transfection [68], magneto-mechanical actuators of cell surface receptors [69], magnetically triggered drug release [70] and magnetic fluid hyperthermia (MFH). In this latter biomedical application, magnetic NPs are placed in contact with cancer tissues after which an alternating magnetic field (AMF) is employed leading to heat dissipation until an adequate thermal dose provokes cell death by different mechanisms [71]. Recently, some authors have demonstrated that one of those mechanisms refers to the generation of ROS [72,73]. Therefore, one potential cancer cell death mechanism might be the ROS production from iron-oxide NPs in combination with AMF. This phenomenon can be related to enhanced kinetic activity of the Fenton-like reaction or to the decreased ability of a cancer cell to scavenge ROS when high temperatures are present in the system [73]. For this reason, hyperthermia as a biomedical application has gained a lot of attention with more than 350 ongoing clinical trials using magnetic NPs and hyperthermia in the USA and Europe [63]. Furthermore, hyperthermia has been also used as an adjuvant treatment to improve anticancer treatments like chemotherapy and radiotherapy and indicates that MFH has a promising role as an adjuvant to chemotherapy by potentiating the effects of anticancer agents.

Interestingly, a recent in vitro study described a sonodynamic-mediated effect by employing low-intensity ultrasound (US) in combination with iron-oxide NPs and demonstrated increased production of ROS. Indeed, it is believed that US exposure, such as that in AMF, facilitates the iron release necessary to trigger the Fenton reaction which ultimately is responsible for generation of ROS [74]. The authors used US with 1 MHz intensity and different iron-oxide NPs concentrations as the sonosensitizer to explore their combined activity on the breast cancer cell line MCF-7. They considered four different groups: cells without any treatment, cells treated with iron-oxide NPs, cells exposed to US and cells treated with the sonodynamic combination of iron-oxide NPs and US. A significant decrease in cell proliferation was observed when MCF-7 cells, in in vitro experiments, underwent the combined treatment with US (at a frequency of 1 MHz with a 5 cm^2^ probe, exposing cells for 1 min to a horizontal beam of continuous US wave at fixed output intensities of 2 W/cm^2^) and iron-oxide NPs compared to the control group, iron-oxide NPs group and US group, suggesting that the sonodynamic effect of US and iron-oxide NPs might be due to ROS generation.

### 2.2. Silver NPs

Among MNPs, silver NPs (AgNPs), ranging between 1 and 100 nm in size, are very attractive due to their remarkable optical, thermal conductivity and electrical properties [46], which support their main role in the industrial applications of photonics, microelectronics and catalysis. In particular, AgNPs have great potential in a broad range of nanomedicine applications as biomedical device coatings, antimicrobial agents, imaging probes, drug-delivery carriers and diagnostic and optoelectronic platforms [75].

Focusing our attention on their antimicrobial activity, AgNP-cytotoxicity is characterized by their ability to release silver ions from their surface when placed in contact with an aqueous environment, as the particle surface dissolves. The release of Ag^+^ ions is affected by several factors including the size and shape of NPs, capping agent and colloidal state. The interaction of silver ions with thiol groups on bacterial cell surface is caused by the large number of sulfur-containing proteins. Therefore, AgNPs can influence bacterial cell viability by their interaction with sulfur-containing proteins into the bacteria cell membrane [76]. Ag^+^ ions work by substituting other essential metal ions, such as Zn^2+^ and Ca^2+^, in critical bacterial enzymes and proteins, provoking damage of cellular respiration and cell death. AgNPs can also anchor to the surface of the bacterial cell wall and penetrate it, causing structural changes to the membrane or increasing its permeability. All of these phenomena lead to cell death. Alongside this, it has been proposed that silver ions, particularly Ag^+^, released from AgNPs can provoke an interaction with DNA phosphorus moieties, causing the inactivation of DNA replication [76]. Furthermore, AgNPs can react with the sulfhydryl groups of metabolic enzymes that belong to the bacterial transport chain of electrons, proving their inactivation.

ROS and free-radical generation are another AgNP mechanism causing a cell-death process, demonstrated in in vitro studies. Indeed, the potent cytotoxic activity of AgNPs and ability to sustain antibacterial, antifungal and antiviral activity and, for some authors, also their anticancer activity [77], is mainly due to their ability to induce ROS and free-radical species production, such as hydrogen peroxide, superoxide anion, hypochlorous acid, hydroxyl radicals and singlet oxygen [78,79]. Regarding their antibacterial role, once the AgNPs are in contact with bacteria, the free radical-mediated pore generation into the cell wall is the leading mechanism of bacterial cell death. Therefore, some authors have investigated how to improve the AgNPs’ antimicrobial activity via localized surface plasmon resonance (LSPR) to increase the ROS generation by exposure to visible light [80].

The LSRP is related to the electron collective oscillation at the metallic structure interface, which can be induced by the electron-magnetic interaction of the metal with light at an appropriate wavelength. In other words, LSPR is an optical phenomenon generated by conductive nanoparticles, smaller than the incident wavelength, interacting with light [81]. In this regard, Vasil’kov and colleagues have published research where they have investigated how antibacterial properties of AgNPs increased when the plasmon resonance effect occurs due to a 470 nm laser radiation application for 5 min, with a power of 5 mW, in a Petri dish containing AgNPs, Gram-positive and Gram-negative bacteria [82]. The main objective of this study was to increase the antibacterial activity of AgNPs by exploiting the capability of LSPR to influence the electronic state of the particles and increase the number of silver ions (Ag^+^) which ultimately are responsible for the antimicrobial effect. Recently, research from da Silva and colleagues [80] has shown a link among AgNPs, LSPR, ROS and the AgNPs’ antimicrobial activity. The authors claim that, even if some studies support the idea that Ag^+^ plays a main role in antibacterial activity [83], together with the capability of AgNPs to introduce nicks in the cytoplasmic membrane [84], AgNPs and Ag^+^ can also induce ROS formation, which damages the cell cytoskeleton, oxidizing nucleic acids and proteins, leading to potential chromosomal aberrations and cell death [85]. In this study, the peculiar association between the bactericidal effect of AgNPs, along with an intracellular ROS increase, probably as generated by LSPR via use of a LED floodlight (50 W) on the AgNPs’ surface, was explained. This finding, in our opinion, seems very promising since it may broaden the opportunity, as we have mentioned before, to also study the cytotoxicity of AgNPs against cancer cells [86,87].

### 2.3. Gold NPs

Another noble metal with great abilities and promising results in medicine is gold (Au) [88]. In particular, gold nanoparticles (AuNPs) have demonstrated anticancer properties derived from different mechanisms which can be explained by varied Au properties. AuNPs can be exploited for anticancer purposes via several approaches, such as drug-delivery, anti-angiogenic, photothermal and photodynamic effects [89].

The AuNPs’ photothermal application is due to the multiplicative effects of increased local absorption of laser radiation at near-infrared (NIR) frequencies by LSPR, inducing hyperthermia in cancer tissue, revolutionizing the traditional and widespread laser hyperthermia of tissues [90]. Additionally, visible light irradiation can allow for hyperthermia by LSPR, as recently demonstrated by Mendes et al., in which 14 nm AuNPs were combined with green laser light despite the fact that therapeutic efficacy of such an approach is limited in cancer due to poor penetration of light through tissue [91,92].

**Figure 3:**
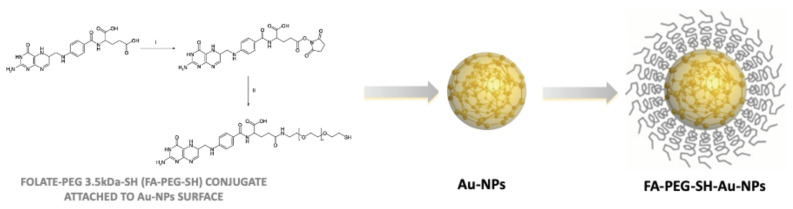
Gold nanoparticles (Au-NPs) can be decorated by adding the folic acid carboxyl group. Folic acid was activated by carboxyl groups by N-hydroxysuccinimide (NHS) and dicyclohexylcarbodiimide in anhydrous dimethyl sulfoxide (DMSO) and conjugation of NHS-ester activated folate to NH_2_-PEG3.5kDa-SH (FA-PEG-SH) in anhydrous DMSO in presence of triethylamine [19].

In this regard, to overcome the low light penetration into tissue, it has also been proved that Au-based nanotherapeutics can absorb radiofrequency (RF) and produce heat, giving the possibility to treat deeply localized tumors by using Au and hyperthermia-based options [93]. More intriguing is the approach shown by Brazzale and colleagues, where targeted AuNPs might be activated and kill cancer cells by US [19] (Figure 3). In this pioneering in vitro work, the role of targeted AuNps as sonosensitizers in SDT, an innovative anticancer approach where, it is generally accepted, a non-toxic molecule or system (chemical actuator), i.e., the sonosensitizer, is activated by US (physical activator), yielding oxidative damage by ROS generation, and consequent cancer cell death [94,95]. More specifically, the authors suggest that US, used at the frequency of 1.866 MHz for a total 5 min of exposure, through a physical phenomenon called sonoluminescence [96], might drive the AuNPs’ plasmonic effect, as derived from LSPR, to be able to convert the photon energy to heat in order to induce cellular damage via ROS production. Analysis of intracellular ROS production was investigated in two different cell lines, HCT-116 and KB, and demonstrated that, while cells incubated with AuNPs in the absence of US and US alone did not encounter an increase in intracellular ROS productions, cells that were exposed to both AuNPs and US, i.e., sonodynamic treatment, were subjected to a significant increase in ROS production and therefore enhanced death rates.

### 2.4. Titanium Dioxide NPs

Titanium dioxide (TiO_2_) is one of the most extensively used nanomaterials for several applications [97], but the photocatalytic properties of TiO_2_NPs have raised many issues as a result of ROS generation while UV irradiation is performed. Indeed, electrons in the TiO_2_ valence band absorb the photon energy under UVA irradiation, and jump to the conduction band, allowing extraction of electrons from water or hydroxyl ions generating hydroxyl radicals by valence band holes. Other methods of ROS formation, such as superoxide anion and singlet oxygen by additionally mechanisms, have also been demonstrated [98,99]. However, the photocatalytic properties of this NP make TiO_2_ a valuable competitor for some biomedical applications, such as in killing microorganisms [100,101] and treating malignant tumors. The latter application has been investigated since 1992, when Cai et al. studied the effect of photoexcited TiO_2_ on cancer cells in in vitro studies [102]. From this initial investigation, other researchers have studied the cytotoxicity by photoexcited TiO_2_ on cancer cells [103], but more intriguing has been two recent scientific works where the TiO_2_NPs have been found to be effective in PTT against a melanoma cancer model and also as sonosensitizer in SDT against a breast cancer model [104,105].

In the first study, the authors assessed the application of PEGylated TiO_2_NPs in inducing hyperthermia and necrosis in in vivo melanoma tumors after PTT consisting of a continuous wave near-infrared (NIR) laser diode at 808 nm wavelength with an intensity of 2 W/cm^2^ for seven minutes. Four mice groups were enrolled in the experiments and the main result showed that in the PEGylated TiO_2_NPs + laser therapy group, not only did the tumor growth cease, but the tumor size also shrank according to the ultrasonography images and the histopathological examination in the three days following the experiment. Interestingly, five mice from the PEGylated TiO_2_NPs + laser therapy group were euthanized after three months of follow-up to demonstrate biocompatibility of these PEGylated TiO_2_NPs. However, no data about the survival rate of those animals were reported.

The latter work investigated TiO_2_NPs, more precisely the spherical carbon-doped titanium dioxide nanoparticles (C-doped TiO_2_NPs), as a sonosensitizer in SDT in order to overcome the major limitation associated with cancer therapies that involve electromagnetic waves, i.e., the shallow penetration depth of light sources into tumor tissue [105]. Taking this into consideration, Yang and colleagues investigated whether C-doped TiO_2_NPs were able to suppress the proliferation of 4T1 breast cancer cell line in both in vitro and in vivo models in combination with US treatment (US frequency of 1.0 MHz and a duty cycle of 50% with a negative pressure of 0.33 MPa and intensity of 1.8 W/cm^2^ for 90 s) in order to inhibit tumor growth. Firstly, in the in vitro study, the authors quantified ROS production between treatment groups and found that C-doped TiO_2_NPs, in combination with US, significantly increased the level of ROS compared to control group. This result corroborated that, under US irradiation, ROS generation could be improved in the presence of C-doped TiO_2_NPs. Thereafter SDT cytotoxicity was evaluated confirming that SDT, i.e., 4T1 cells cultured with C-doped TiO_2_NPs and subjected to US exposure, induced higher cytotoxicity in 4T1 cells than the other treatment groups. Interesting was the speculation about the possible pathway of cell damage induced by SDT, where the authors suggested a role of sonoluminescence in the C-doped TiO_2_NPs activation to generate more ROS and kill 4T1 breast cancer cells [96]. Finally, in this work, to further investigate the cell death of 4T1 cells induced by SDT, an in vivo study was performed. Groups of 5 nude mice were enrolled, all bearing subcutaneous 4TI breast cancer cells, and the data showed that the C-doped TiO_2_NPs group (150 mg/mL C-doped TiO_2_NPs at day 0 and day 7) and US group (PBS at day 0 and day 7) could not suppress the tumor growth, while the SDT group (150 mg/mL C-doped TiO_2_NPs at day 0 and day 7 with US exposure) was able to significantly delay tumor growth in that the relative tumor volume at endpoint was almost half that of other control groups. Moreover, by using histologic staining of the tumor site, authors observed that SDT enhanced the ability to cause 4T1 cell death compared to the other groups, confirming that C-doped TiO_2_NPs could be considered as sonosensitizers for sonodynamic treatments, and in general as an efficient strategy for alternative cancer treatments.

### 2.5. Zinc Oxide NPs

Relying on these promising results from TiO_2_NPs as photo- or sonosensitizer in PPT and SDT respectively, ZnONPs have also been investigated as photo- or sonosensitizers for cancer therapy. Indeed, the ZnO electronic structure is part of the semiconducting metal oxide family like TiO_2_ and defines ZnONPs’ catalytic properties [106]. Therefore, ZnONPs can be photoexcited in the UV-A range (315 ≤ λ < 400 nm) [107]. Another possibility to excite ZnONPs in aqueous solutions is the application of US, as propagation in a liquid milieu causes the well-known physical phenomenon called acoustic cavitation. A number of secondary effects are determined by this phenomenon, and in particular sonoluminescence, which represents a sonoluminescent emission, mainly a UV light, due to the cavitation bubble implosion [96]. For this reason, many researchers suggest that ZnONPs bring about cytotoxic effects when exposed to US via generation of ROS as their principal mechanism of bacterial and cancerous cell death [108].

Through investigating the antibacterial activity of ZnONPs in vitro studies, it is well known that ZnONPs have antimicrobial activity against Gram-positive (*B. cereus, B. subtilis*, *E. faecium, L. monocytogenes*, *S. aureus* and *S. epidermis*) and Gram-negative (*E. coli, K. pneumoniae*, *P. aeruginosa* and *Salmonella sp.*) bacteria [109]. Furthermore, Seil and colleagues have shown that intrinsic antibacterial activity against *S. aureus* of ZnONPs might be enhanced, up to 76%, by US exposure as this provides an additional mechanism to decrease bacterial activity via enhanced generation of hydrogen peroxide by *S. aureus* compared to control samples [110].

Recently Racca et al. [111] reported a novel study demonstrating the highly efficient killing capability of amino-propyl functionalized ZnO nanocrystals (ZnONCs) in association with high-energy shock waves (HESW) for cancer cell treatment. This in vitro work investigated KB cells’ viability after incubation with a non-toxic concentration of ZnONCs (10 μg/mL) in combination with HESW exposure. To demonstrate the effective synergy between the HESW and ZnONCs, cells were pre-incubated for 24 h with 10 μg/mL ZnONCs and then exposed to HESW (by considering several energy flux density ranges, for instance 0.15–0.22–0.3-0.4-0.52 mJ/mm^2^, corresponding to positive peak pressures of 29.1, 39.4, 50.3, 61.7 and 74.1 MPa, respectively). Authors observed that a single treatment was not able to induce a significant difference in cell viability between HESW exposure alone and ZnONCs + HESW exposure. In contrast, multiple HESW treatments (3 times/day) showed cytotoxicity only for those cells pre-incubated with ZnONCs. Studies of the mechanism showed that the ROS role was controversial. Therefore, the authors suggested that the anticancer activity was due to a combination of several effects, including the non-inertial cavitation, the so- called “nanoscalpel effect,” as well as an imbalance of electric change, involving the ZnO piezoelectric behavior [111].

## 3. Conclusions

Metal-based NPs are highly demanded because they have broad range of applications in healthcare, cosmetics and industry. Particularly, in this review, authors have focused their attention on novel biomedical applications of IONPs, AgNPs, AuNPs, TiO_2_NPs and ZnONPs in photodynamic, photothermal and sonodynamic therapies. Specifically, these MNPs are able to be triggered by different physical actuators to generate ROS for the selective killing of bacteria and cancer cells. Their pivotal role as remotely activated NPs for therapeutic ROS generation has been highlighted for IONPs under magnetic field, and for AgNPs, AuNPs, TiO_2_NPs and ZnONPs under light or US exposure.

Despite these promising results, MNPs’ potential druggability requires further extensive evaluation before they can reach clinical applications. Therefore, future research involving MNPs should consist of robust pre-clinical studies with a predominant focus on acceleration of their clinical translation for biomedical uses.

## Figures and Tables

**Table 1:** Main advantages and disadvantages of metal-based nanoparticles (NPs).

	Advantages	Disadvantages
**Metal-Based NPs**	Biocompatibility High oxidation efficacy High photostability High binding affinity Low cost Surface enhanced Raman scattering Strong plasma absorption Biological system imaging Determine chemical information on metallic nanoscale substrate	Instability Impurities loaded during their synthesis Difficulty in synthesis Thermal decomposition

## Data Availability

No new data were created or analyzed in this study.
